# A case of acute obstructive suppurative pancreatic ductitis complicated with acute cholangitis diagnosed only after the removal of a pancreatic duct stent

**DOI:** 10.1002/deo2.352

**Published:** 2024-03-21

**Authors:** Hirotaka Oura, Harutoshi Sugiyama, Takayoshi Nishino

**Affiliations:** ^1^ Department of Gastroenterology Tokyo Women's Medical University Yachiyo Medical Center Chiba Japan

**Keywords:** acute cholangitis, alcoholic pancreatitis, chronic pancreatitis, endoscopic retrograde cholangiopancreatography, pancreatic ductitis

## Abstract

Acute obstructive suppurative pancreatic ductitis (AOSPD) is a rare complication of chronic pancreatitis that presents with high fever and abdominal pain. A 63‐year‐old man underwent plastic bile duct stent and plastic pancreatic duct stent (PDS) placement for benign stricture in the intrapancreatic bile and pancreatic ducts associated with chronic pancreatitis; the stents were routinely replaced. Seven months after the last replacement, the patient presented to our hospital with dark urine but without fever or abdominal pain. Subsequent blood tests revealed elevated levels of hepatobiliary enzymes, white blood cells, and C‐reactive protein. However, the pancreatic enzyme levels remained unchanged, and abdominal computed tomography showed the absence of inflammation around the pancreas. He was initially diagnosed with acute cholangitis (AC) due to bile duct stent dysfunction and subsequently underwent emergency endoscopic retrograde cholangiopancreatography. As obstruction of the PDS was suspected, both bile duct stent and PDS were replaced. Although the collected bile did not exhibit purulence, a white purulent fluid was released after replacing the PDS. Cultures from the bile and pancreatic exudates revealed the presence of *Klebsiella oxytoca*. Consequently, the patient was diagnosed with AOSPD and AC. In this patient, endoscopic retrograde cholangiopancreatography was performed after the diagnosis of AC alone; however, relying solely on AC treatment might not have ameliorated the patient's condition. The patient did not complain of any abdominal pain and was diagnosed with AOSPD only after the replacement of his PDS. Our case suggests that AOSPD may be a pitfall in the identification of the source of inflammation in patients with chronic pancreatitis.

## INTRODUCTION

First documented in 1995, acute obstructive suppurative pancreatic ductitis (AOSPD) represents a unique manifestation characterized by the “suppuration of the pancreatic duct without the development of a phlegmon, pseudocyst, or contiguous pancreatic abscess”.^1^ AOSPD, albeit rare, emerges as a complication of chronic pancreatitis (CP) and usually presents with high fever and abdominal pain.^2^ Recently, a male patient presented at our hospital with a complaint of darkened urine. Despite the absence of fever or abdominal pain, laboratory tests revealed elevated C‐reactive protein (CRP) levels. Notably, emergency endoscopic retrograde cholangiopancreatography (ERCP) was performed under the initial diagnostic impression of acute cholangitis (AC). Subsequent analysis, however, led to the final diagnosis of AOSPD complicated by AC.

## CASE REPORT

**FIGURE 1:**
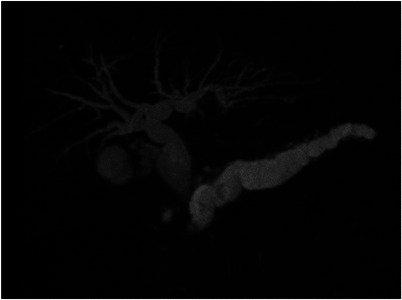
Magnetic resonance imaging performed when the patient initially visited our hospital 9 years ago. Intrapancreatic bile duct stricture, pancreatic duct stricture in the pancreatic head, and distal pancreatic duct dilation were observed.

A 63‐year‐old man had been diagnosed with alcoholic chronic pancreatitis (CP) 9 years ago and regularly visited our hospital. Magnetic resonance imaging conducted during the patient's initial visit revealed intrapancreatic bile duct stricture, pancreatic duct stricture in the pancreatic head, and distal pancreatic duct dilation (Figure 1). Five years prior to presentation, the patient underwent plastic pancreatic duct stent (PDS) placement to address a benign stricture in the main pancreatic duct, prompted by persistent pancreatic abdominal pain. Six months later, the patient developed cholangitis due to an intrapancreatic bile duct stricture, necessitating the placement of a plastic bile duct stent (BDS). Subsequently, the 7Fr‐BDS and 7Fr‐PDS were exchanged regularly. Despite the outpatient physician's recommendation for periodic replacements at least every 3 months, financial constraints triggered the patient to request an extension of the intervals between replacements. Given this circumstance, the outpatient physician conscientiously explained the risks associated with prolonged stent placement, emphasizing the potential for obstruction. The patient was advised to seek immediate medical attention at our hospital, even if only mild symptoms were present.

**FIGURE 2:**
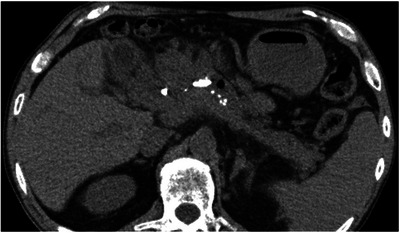
Abdominal computed tomography image at the time of consultation when the patient presented at the hospital. Atrophy and calcification of the pancreatic parenchyma associated with chronic pancreatitis were noted. The fat levels surrounding the pancreas remained unchanged.

**FIGURE 3:**
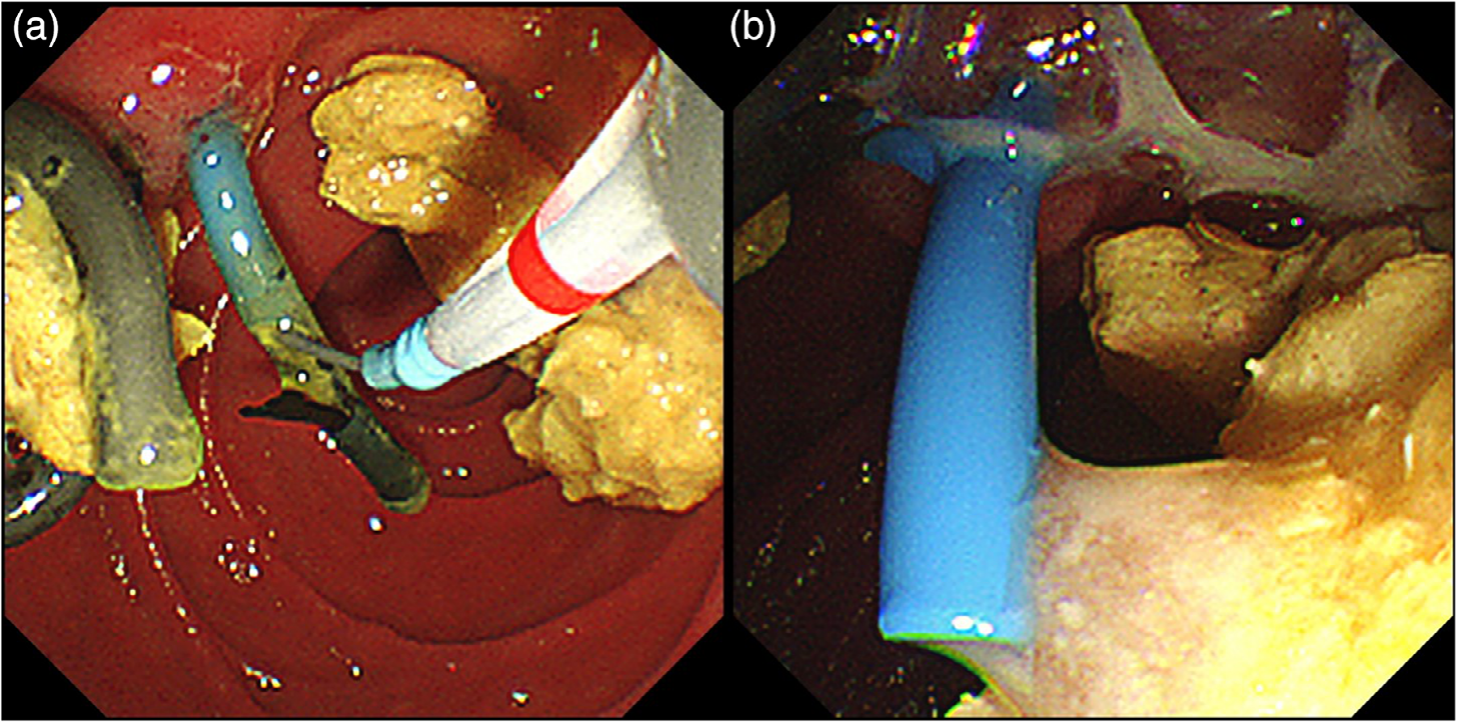
Endoscopic images taken during emergency endoscopic retrograde cholangiopancreatography. (a) Obstruction of the bile duct stent and the pancreatic duct stent was suspected. (b) A substantial amount of white purulent fluid was released when the pancreatic stent was replaced, while the collected bile was not purulent.

**FIGURE 4:**
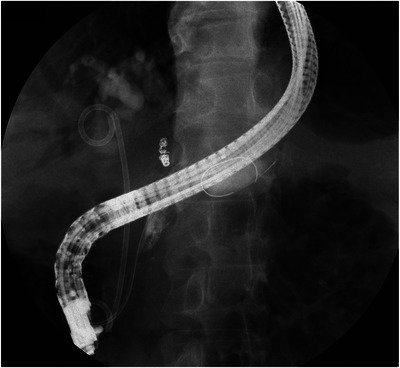
Pancreatography images obtained during emergency endoscopic retrograde cholangiopancreatography. The patient presented with pancreatic duct stenosis near the pancreatic head and dilatation of the caudal pancreatic duct.

The patient presented at our hospital with a chief complaint of dark‐colored urine with no apparent abdominal pain. Seven months had passed since the last scheduled replacement. The patient, diagnosed with type 2 diabetes mellitus, received insulin therapy and consumed approximately 50 g of alcohol daily. Upon presentation, his temperature was 36.7°C, and other vital signs remained normal. Physical examination revealed a flat, soft, and tender abdomen. Blood tests showed elevated levels of aspartate aminotransferase (72 U/L), alanine aminotransferase (35 U/L), alkaline phosphatase (298 U/L), and γ‐glutamyl transpeptidase (253 U/L). The total bilirubin concentration was 0.9 mg/dL. Renal dysfunction was evident with a blood urea nitrogen level of 46.9 mg/dL and a creatinine (Cre) level of 2.35 mg/dL. The C‐reactive protein (CRP) level and white blood cell (WBC) counts were relatively high (7.66 mg/dL and 13.25×10^3^, respectively). Moreover, the procalcitonin concentration was 27.27 ng/mL, suggesting bacterial infection. The pancreatic enzyme levels were within normal limits, with amylase and lipase levels of 43 and 19 U/L, respectively. Abdominal computed tomography revealed pancreatic parenchymal atrophy and calcification, but no inflammation was observed around the pancreas (Figure 2). In addition to the elevated CRP levels on blood tests and abnormal findings on liver function tests, BDS dysfunction was suspected, considering the prolonged interval of 7 months since the last replacement; hence, the patient was diagnosed with AC. The Cre concentration, which was initially within the normal range at the previous outpatient visit, was elevated at this point. Given the diagnosis of Grade III severe AC accompanied by renal dysfunction, emergency ERCP was performed. In addition, the PDS and BDS appeared obstructed (Figure 3a), thus necessitating their replacement. Although the biliary sludge was drained upon BDS removal, the collected bile was not purulent. However, upon PDS replacement, a substantial amount of white purulent fluid was released (Figure 3b and Video S1). Cultures from both bile and pancreatic exudates identified the presence of *Klebsiella oxytoca*. Blood culture tests yielded negative results. Pancreatic ductography performed during emergency ERCP revealed a pancreatic duct stricture in the pancreatic head and dilatation of the caudal pancreatic duct (Figure 4).

The patient was diagnosed with AOSPD complicated by AC and was treated with meropenem. Blood tests performed on the following day showed improvements in hepatobiliary enzyme levels, renal function, and CRP levels. Upon the patient's request for early discharge, a course of antibiotics was administered for 5 days. Consequently, the patient was discharged, without clinical symptoms. Three months after discharge, the patient continued to visit the outpatient clinic without evident problems.

## DISCUSSION

Although no fatal cases have been reported, AOSPD can lead to sepsis^3^ and is a rare but important complication of CP. The reported causes of pancreatic duct obstruction in AOSPD include pancreatic duct obstruction due to CP, pancreatic cancer, and/or pancreatic surgery.^1^, ^2^, ^3^, ^4^, ^5^ AOSPD is usually accompanied by fever and abdominal pain. In a retrospective review of 20 patients with AOSPD, a temperature of 37.5°C or higher was observed in 14/20 patients (70%), while abdominal pain was observed in 19/20 (95%).^2^ By contrast, there was a report of an asymptomatic patient who did not complain of abdominal pain.^6^ In the present case report, the patient did not present with fever or abdominal pain. The primary reason for his visit was the darkening of urine. However, blood tests did not indicate an increase in bilirubin levels, which was assumed to be due to dehydration associated with the infection. The outpatient physician, recognizing the potential risk of stent obstruction due to prolonged placement, advised the patient to promptly seek medical attention when minor symptoms occur, such as dark urine, fatigue, or loss of appetite. This preemptive patient education aims to facilitate early treatment before the typical symptoms of AOSPD manifest, such as abdominal pain and fever. As obstruction of the BDS was suspected, AC was initially diagnosed as the primary focus of inflammation, leading to the performance of emergency ERCP. The Tokyo Guidelines (outline the following criteria for diagnosing AC: (1) systemic inflammation, (2) cholestasis, and (3) bile duct lesions on imaging examination.^7^ The patient demonstrated elevated CRP levels, abnormal liver function test results, and signs of BDS obstruction, leading to a diagnosis of AC, fulfilling the criteria for the Tokyo Guidelines. In reality, the bile culture yielded a positive result and, therefore, confirmed the diagnosis of AC. However, the PDS was completely occluded, and the AOSPD was complicated. Furthermore, the bile collected during endoscopic examination did not exhibit purulence, while the pancreatic exudate appeared whitish and purulent. Therefore, the high WBC and CRP concentrations at the time of the patient's visit were attributed to AOSPD. Consequently, treatment focused solely on AC may not have effectively addressed the patient's general condition.

In our case report, the patient did not complain of abdominal pain, and both blood tests and imaging findings ruled out pancreatitis or abscess formation. Consequently, the pancreas was not initially suspected as the focal point of inflammation. As a result, the diagnosis of AOSPD was confirmed only after the PDS was replaced. Endoscopic stent placement for symptomatic main pancreatic duct stenosis is recommended by European guidelines, and the most common complications associated with plastic stent placement are mild pancreatitis and worsening abdominal pain (average occurrence, 6.2%; range, 4%‐39%).^8^ No incidence of pancreatic ductitis associated with the long‐term placement has been reported. Our case report underscores the potential diagnostic challenge of AOSPD in identifying the inflammatory foci in patients with CP, especially in those with prolonged PDS placement. In addition, this case report emphasizes the necessity of considering the possibility of stent‐obstructing pancreatic ductitis, warranting early endoscopic treatment even in the absence of typical symptoms.

## CONFLICT OF INTEREST STATEMENT

None.

## INFORMED CONSENT

Informed consent was obtained from the patient for this case report.

## Supporting information


**Video S1** Emergency endoscopic retrograde cholangiopancreatography was performed based on the diagnosis of acute cholangitis with elevated C‐reactive protein levels without fever or abdominal pain. When the pancreatic duct stent was replaced, a substantial amount of white purulent fluid was released, leading to the diagnosis of acute obstructive suppurative pancreatic ductitis.
Video text
A plastic bile duct stent (BDS) and pancreatic duct stent (PDS) were inserted to address a benign stricture in the intrapancreatic bile and pancreatic ducts associated with chronic pancreatitis.
The patient was initially diagnosed with acute cholangitis (AC) due to dysfunctional BDS, and emergency endoscopic retrograde cholangiopancreatography (ERCP) was performed.
Given the suspicion of obstruction in the PDS, both BDS and PDS were replaced.
The PDS was replaced through the guidewire first.
During the PDS replacement, a substantial amount of white purulent fluid was released.
The bile collected was not purulent.
Cultures from the bile and pancreatic exudates revealed the presence of *Klebsiella oxytoca*.
Subsequently, the diagnosis was confirmed as acute obstructive suppurative pancreatic ductitis combined with AC.
